# Biologically responsive carrier-mediated anti-angiogenesis shRNA delivery for tumor treatment

**DOI:** 10.1038/srep35661

**Published:** 2016-10-19

**Authors:** Junyi Che, Anqi Tao, Shun Chen, Xiaoming Li, Yi Zhao, Weien Yuan

**Affiliations:** 1 School of Pharmacy, Shanghai Jiao Tong University, Shanghai, People’s Republic of China

## Abstract

Small interfering RNA (siRNA) has increased the hope for highly-efficient treatment of gene-related diseases. However, the stable and efficient delivery of therapeutic nucleic acids is a prerequisite for the successful clinical translation of RNA interfering therapy. To achieve this, we condensed the low molecular weight polyethyleneimine (PEI, Mw < 2000) with 2,6-pyridinedicarboxaldehyde (PDA) to synthesize a biologically responsive and degradable cationic polymer (abbreviated to PDAPEI) which was utilized as a gene vector for the delivery of a VEGF-A shRNA expression plasmid DNA (pDNA). The resulting electrostatic interaction between PDAPEI and pDNA led to the self-assembly of nanoscale polyplexes with suitable particle size and stable zeta potential. The PDAPEI/pDNA polyplexes demonstrated an outstanding gene transfection and silencing efficiency at 30 w/w ratio, as well as negligible cytotoxicity. Also, the designed polymer showed no stimulation to the innate immune system. Moreover, compared with PEI 25 KDa, the polyplexes accomplished comparatively better anti-angiogenesis efficacy, which resulted in the inhibition of tumor growth in subcutaneous tumor mice models. In conclusion, PDAPEI has great potential to be a gene delivery vector for cancer therapy.

The process of tumor angiogenesis is generally divided into two phases: non-vascular phase and vascular phase^1^. In non-vascular phase, the volume of tumor is less than 2–3 mm^2^, followed by the vascular phase as the tumor continuously grows. During this phase, the vascular endothelial growth factor (VEGF) is overexpressed in tumor cells, which contributes to the hypersecretion of VEGF^2^,^3^. Overexpression of VEGF is regarded as the primary reason for tumor angiogenesis^4^,^5^. The VEGF may have a positive effect on the migration of endothelial cells through paracrine, which is responsible for the tube formation. More importantly, the abnormally rapid growth of solid tumors is mainly dependent on a large amount of nutrition supplied by blood vessels. Furthermore, the tumor cells can pass through blood vessels or lymphatic tissues, which lead to the invasion and metastasis of the tumor^6^,^7^,^8^. Therefore, inhibition of the VEGF expression has become a key way to restrain tumor growth and metastasis.

Despite the efforts in cancer treatment, mortality and relapse rates of cancer remain high^9^,^10^. However, RNA interference (RNAi) brings new approach for cancer treatment. Small interfering RNA (siRNA, double stranded RNA molecules with 21~25 bp length) is used to direct homology-dependent control of gene activity^11^,^12^,^13^. Even disease-specific alleles that differ from normal allele by only one or few nucleotide substitutions are permitted to target. Moreover, designed siRNA molecules could inhibit the expression of the target gene at very low concentration^11^.

In order to address this issue, direct delivery of an adequate amount of siRNA to target cells by designed viral vectors or non-viral vectors has been reported in the field of gene delivery^14^,^15^. Among all the available vectors, polycationic carrier and cationic lipid are the most popularly employed or used vectors. Cationic lipid is able to resemble traditional pharmaceuticals with little immunogenicity, and has no potential for viral infection. Nevertheless, cationic lipid is limited for clinic applications because of its inevitable toxicity and low transfection efficiency^16^,^17^. Compared with other non-viral vectors, polycationic carrier is attractive for its better biocompatibility, lower immunogenicity and easier modification^18^. Polycation can condense nucleic acid materials into nanoparticles through electrostatic interaction, helping endosomal escape of nucleic acid materials by proton sponge effect and then release nucleic acid in the cytoplasm^19^. The materials for nucleic acid delivery can further transcribe to shRNA in the nucleus and enter the cytoplasm, producing siRNA by the RNase III endonuclease Dicer. The Dicer hands off the siRNA to RNA-induced silencing complex (RISC), which combines with the target mRNA by intermolecular base pairing^20^,^21^.

In this study, we envisaged to use siRNA to silence the VEGF-A gene for the purpose of inhibition of tumor angiogenesis. We chose cationic polymer as the vector and constructed a siRNA expression plasmid DNA to supply a certain amount of siRNA. Polyethylenimine (PEI) consisting of repeating units of amine group and -CH_2_CH_2_- group is a representative polycationic vector, whose transfection efficiency increases with the increase of molecular weight, while accompanied by higher cytotoxicity^22^,^23^. To solve these problems, a safe and effective gene vector is required to achieve intracellular bioactivity. Therefore, we combined the positive charged PEI (any of Mw < 2000) with PDA to obtain a new vector, PDAPEI, for the targeted delivery of plasmid to tumor cells (Fig. 1). Previous studies demonstrated that PDA cross-linked with PEI could metabolize itself into low-molecular-weight PEI of negligible cytotoxicity and PDA in the acidic environment^24^,^25^.

In this work, the physicochemical characteristics of PDAPEI including DNA binding ability, particle size, zeta potential and morphology were investigated respectively in details. Furthermore, gene transfection efficiency and cytotoxicity mediated by PDAPEI-pDNA polyplexes formed of different mass ratios were investigated *in vitro* to optimize the mass ratio (w/w). Meanwhile, the inhibition of VEGF-A expression was measured *in vitro*. For *in vivo* experiments, the anti-tumor efficacy of PDAPEI-pDNA polyplexes in subcutaneous tumor models and the microvessel density in tumors were explored to certify their anti-angiogenesis ability in cancer therapy. Moreover, the *in vivo* cytotoxicity was evaluated by histological examination, and the innate immune response was further investigated by ELISA.

## Methods

### Materials

PEI 1800 and 25 kDa were purchased from Sigma-Aldrich. Anhydrous ethylene dichloride was obtained from Sigma-Aldrich. 2,6-pyridinedicarboxaldehyde (PDA) was purchased from TCI (Shanghai) Development Co., Ltd. Cellulose membranes (MWCO = 10,000 Da) were purchased from Thermo Scientific. Water was purified using a milli-Q instrument (Millipore). Poly (ethylene glycol) (PEG) standards kit (ranging from 106 to 20100 Da in molecular weight) was purchased from Polymer Standards Service GmbH. All the reagents were used without further purification.

Plasmid DNA encoding GFP and mouse-VEGF-A siRNA expression vector was constructed from pGPU6/GFP/Neo vector (Bioroot Biology, Shanghai, China) according to a previous report. The targeted VEGF-A sequences were 5′- CGATGAAGCCCTGGAGTGC -3′. And the plasmid was amplified using EndoFreeTM Plasmid Maxi (Qiagen). High-capacity cDNA reverse transcription kits were purchased from Applied Biosystems. RNeasy Mini Kit was purchased from Qiagen. The primers were supplied from Biotnt (Shanghai).

### Synthesis of the polymer

**Figure 2:**
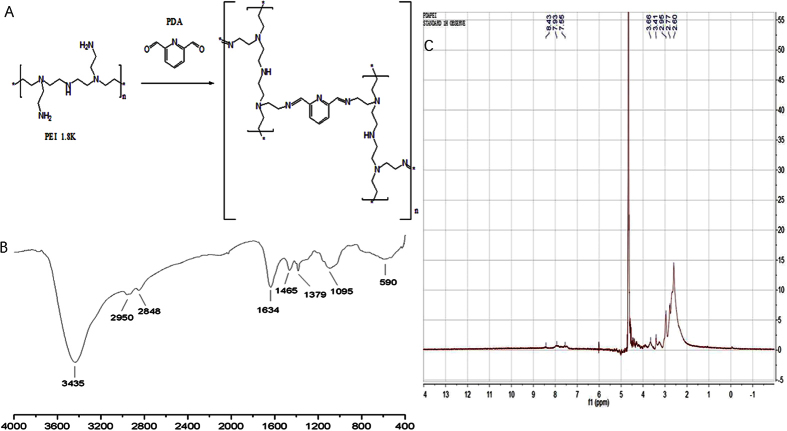
(**A**) Synthesis reaction of PDAPEI. (**B**) IR of PDAPEI. (**C**) NMR of PDAPEI.

The synthesis method was referred to the previously reported method^25^,^26^,^27^,^28^,^29^,^30^,^31^,^32^. Briefly, 1 mmol PEI 1.8 k was introduced in 20 mL anhydrous ethylene dichloride (EDC) solution and stirred vigorously to dissolve^25^,^26^. 2 mmol PDA was dissolved in 20 mL anhydrous EDC, then added dropwisely into PEI solutions with vigorous stirring at room temperature. After stirring for 48 h, the solution was evaporated to remove the solvent. The viscous residue was dissolved again in deionized water and dialyzed using a cellulose membrane of MWCO 10,000 Da for additional 24 h. The yellowish product was obtained after 2 days of lyophilization. The product was named as PDAPEI (Fig. 2).

### Preparation of PDAPEI/pDNA polyplexes

PDAPEI/pDNA polyplexes were prepared at various weight/weight (w/w) ratios. In order to obtain specified w/w ratios of PDAPEI and pDNA, pDNA was diluted with deionized water (20 ng/μl) and PDAPEI was dissolved with deionized water (2 mg/ml) as stock solutions. Then, PDAPEI stock solution was diluted to different concentrations and added rapidly to pDNA stock solution, followed by pipetting to induce self-assembly of polyplexes. The samples were incubated at room temperature for 30 min. The w/w ratios we chose were 10, 20, 30, 40 and 50, respectively. Similarly, PEI/pDNA polyplexes were prepared as a control.

### Cell culture and animals

Mouse colon adenocarcinoma (CT26) cells were purchased from Cell Bank of Chinese Academy of Sciences (Shanghai, China). CT26 cells were incubated in RPMI 1640 medium containing 10% FBS and 1% antibiotics at 37 °C with the CO_2_ concentration at 5%.

Healthy BALB/c mice (female, 5-week old, weight 20 ± 2 g) were purchased from Institute of Zoology, Chinese Academy of Sciences (Beijing, China). All animals survived under Specific Pathogen Free (SPF) environment in the laboratory animal facility in the school of pharmacy, Shanghai Jiao Tong University. The animals were maintained at a relative humidity of 50 ± 20%, noise level under 60 decibels, 12-hour light/dark cycle, and at the temperature range from 20 °C to 28 °C. All animal experiments were conducted strictly in accordance with the guidelines approved by the Regulations for the Administration of Affair Concerning Laboratory Animals for Shanghai Jiao Tong University, the National Institutes of Health Guide for Care and Use of Laboratory Animals (GB14925-2010) and the Regulations for the Administration of Affairs Concerning Experimental Animals (China, 2014).

### Characterization of polyplexes

#### Agarose gel electrophoresis

The condensation ability of PDAPEI was measured by agarose gel electrophoresis assay. 1.5 μl DNA stock solution (20 ng/μl) was mixed with the PDAPEI solutions at different w/w ratios. Then 2 μl loading buffer (30 mM EDTA, 36%(v/v) Glycerol, 0.05%(w/v) Xylene Cyanol FF, 0.05%(w/v) Bromophenol Blue) was added and mixed well. Finally, the mixtures were electrophoresed on the 1% (w/v) agarose gel with TAE running buffer at 120 V for 45 min. The visualization of DNA was imaged using Tanon-3500 Gel Imaging System. Naked DNA was served as a control.

The stability of PDAPEI/pDNA polyplexes was measured in 10% serum. Generally, the PDAPEI and pDNA were mixed at the w/w ratios from 10 to 50 for 30 min at the room temperature. Then, 10% serum was added and the mixtures were incubated at the room temperature for extra 4 h. PEI 25 kDa/pDNA at the w/w ratio of 2 was served as the positive control. The samples were measured by agarose gel electrophoresis assay described above.

### Particle size, zeta potential and morphology measurements

The particle size and zeta potential of PDAPEI/pDNA polyplexes were measured at different w/w ratios by Particle-Size Analyzer (Brookhaven Instruments) and Zeta Potential Analyzer (Zetasizer Nano, Malvern Instruments), respectively at 25 °C. The morphology of PDAPEI/pDNA polyplexes was imaged by transmission electron microscopy (JEOL JEM 2010 system) at the w/w ratio of 2.

### *In vitro* cell transfection

The pDNA encoding green fluorescence protein (GFP) was used to determine transfection efficiency of PDAPEI/pDNA polyplexes. CT26 cells (5–10 × 10^4^/ml) were seeded in 48-well plates and cultured in 500 μl RPMI 1640 medium containing 10% FBS and 1% antibiotics at 37 °C with the CO_2_ concentration at 5%. When each well reached 80–90% confluence, the medium was replaced by 50 μl PDAPEI/pDNA polyplex solutions at different w/w ratios (from 10 to 50, the mass of pDNA was 0.5 μg each well) and 250 μl RPMI 1640 medium (without serum and with 10% serum) for 4 h. Then, the medium was replaced by 500 μl fresh RPMI 1640 medium containing 10% FBS and incubated for extra 44 h. Naked pDNA was used as the negative control. PEI 25 kDa/pDNA at the w/w ratio of 2 was used as the positive control. Phosphate buffered saline 1× served as the blank control. Flow cytometer (BD FACSCalibur) was utilized to quantify the percentage of GFP positive cells.

### *In vitro* cytotoxicity

*In vitro* cytotoxicity of polyplexes against CT26 cells was measured by Cell Counting Kit-8 (CCK) reagent. CT26 cells (1 × 10^4^/well) were seeded into 96-well plates and incubated for 24 h. 10 μl PDAPEI/pDNA solutions at different w/w ratios (from10 to 50) were added into each well for 4 h incubation. Then, 10 μl CCK8 reagent was added into each well and incubated for extra 2 h. Absorbance at 450 nm was measured with multifunctional microplate reader (SpectraMax M3 Multi-Mode Microplate Reader) and reference at 630 nm. PEI 25 kDa/pDNA solution was prepared as the control. The blank control group was considered as 100% cell viability. Cell viability was calculated by [Sample group(OD450)-Sample group(OD630)/Blank group(OD450)-Blank group (OD630)] × 100%.

### Intracellular uptake

To further observe the cellular uptake of PDAPEI/pDNA polyplexes, intracellular localization of PDAPEI/pDNA polyplexes in CT26 cell line was investigated using Confocal Laser Scanning Microscope (CLSM). Plasmid was labeled with the fluorophore Cy5 using the Label IT kit (Mirus, Madison, WI) according to the manufacturer’s instructions. CT26 cells (1 × 10^4^/dish) were seeded into 14 mm glass-bottom culture dishes and cultured for 24 h. The medium was replaced by 500 μl PDAPEI/pDNA polyplexes solution (W/W ratio = 30) and 2500 μl RPMI 1640 medium (without serum) for 4 h. Then, cells were washed twice with PBS to remove non-uptaken polyplexes. Lyso Tracker Green (50 nM) was added to stain the lysosomes for 30 min at 37 °C, washed twice with PBS. Finally, cells were stained with DAPI (1 ug/ml) for 15 min at 37 °C and imaged by CLSM.

### *In vitro* gene silence experiment

CT26 cells were seeded into 6-well plate (1.2 × 10^6^ each well) and incubated at 37 °C for 24 h. Then, cells were treated with PDAPEI/pDNA polyplexes for 4 h. Naked pDNA was used as the negative control and PEI 25 kDa/pDNA prepared at the mass ratio of 2 was used as the positive control. After incubation for 48 h, total RNA was extracted from the cells using RNeasy^®^Mini Kit (Qiagen, Valencia, CA) and transcribed into cDNA using High-Capacity cDNA Reverse Transcription Kit (Applied Biosystems). The cDNA was subjected to PCR amplification using the mus-VEGF-A specific primer (forward: 5′-GAAAGGCTTCAGTGTGGTC-3′; reverse: 5′-CAGGAAT GGGTTTGTCGT-3′). The qPCR was carried out using StepOnePlus™ Real -Time PCR System (Applied Biosystems) for 40 cycles each of which consisted 95 °C for 15 s and 60 °C for 30 s. Meanwhile, expression of GAPDH mRNA was used as an endogenous control. The data was recorded with StepOne software. Finally, the qPCR products were electrophoresed on the 1% agarose gel and stained with EB for semi-quantitative measurements.

### *In vivo* anti-tumor treatment

#### *In vivo* subcutaneous tumor model and treatment

*In vivo* subcutaneous tumor model was set up to evaluate anti-tumor efficacy of PDAPEI/pDNA polyplexes. To establish CT26 tumor-bearing mice models, 0.1 ml of CT26 cell suspension (5 × 10^6^/ml) was injected into the back region of the mice. The mice were maintained in the SPF environment for one or two weeks. When the volume of tumor reached 150 mm^3^ to 200 mm^3^, the mice were randomly divided into 4 groups (6 mice per group): saline, naked pDNA, PEI 25 kDa/pDNA and PDAPEI/pDNA. For treatment groups, 0.1 mL solutions containing 10 μg pDNA were injected intratumorally to the mice. Then the volume of the tumors was monitored by measuring the longest (L) and shortest (W) diameters using a vernier caliper. The volume was calculated using the formula of V = W^2^ × L/2. After 13 days, mice were executed by cervical dislocation and the tumors were removed, weighed and analyzed.

### Calculation of microvessel density

For *in vivo experiments*, the mechanism of anti-tumor effect was assessed by CD31 immuno-histochemical staining. At the 13rd day, six mice of each group were sacrificed to harvest tumor tissues. Tumors were fixed in 10% formalin, dehydrated using a graded ethanol series, embedded in paraffin blocks and sectioned into 4 μm thick slice. Next, tumor sections were stained with rabbit anti-CD31 antibodies to mark microvessels. Five regions of each section for one mouse were chosen randomly to count capillary under the optical microscope for calculating capillary density. The average number of microvessels was recorded as the value.

### *In vivo* cytotoxicity

*In vivo* cytotoxicity of the polyplexes was evaluated by histological examination. Organs recovered from necropsy were fixed in 4% paraformaldehyde, sectioned and stained with hematoxylin and eosin (HE). After staining, the slides were observed and imaged using an optical microscope.

### Innate immune response

For measuring the stimulation of the innate immune system, murine RAW264.7 macrophage cells were seeded on a 12-well plate and cultured in DMEM with 10% FBS. After 48 h, cells were treated with naked DNA, PDAPEI polyplexes and PEI polyplexes without serum for 4 h. Then, the treated cells were incubated in DMEM containing 10% FBS for 24 h. The cell-free supernatants were collected and analyzed using Mouse IL-6 ELISA kit (Dakewe Biotech; DKW12-2060-096; China) and Mouse TNF-α ELISA kit (Dakewe Biotech; DKW12-2060-096; China). The IL-6 and TNF-α level from the serum of Balb/c mice at 6 h after the subcutaneous injection of naked DNA, PDAPEI polyplexes and PEI polyplexes were measured using the same ELISA kit.

### Statistical analysis

The data collected were presented as mean ± standard deviation (S.D.) from several separate experiments. Statistical analysis was tested by one-way ANOVA and a value for *P < 0.05, **P < 0.01 or ***P < 0.001 was considered statistically significant.

## Results and Discussion

### Agarose gel electrophoresis

**Figure 3:**
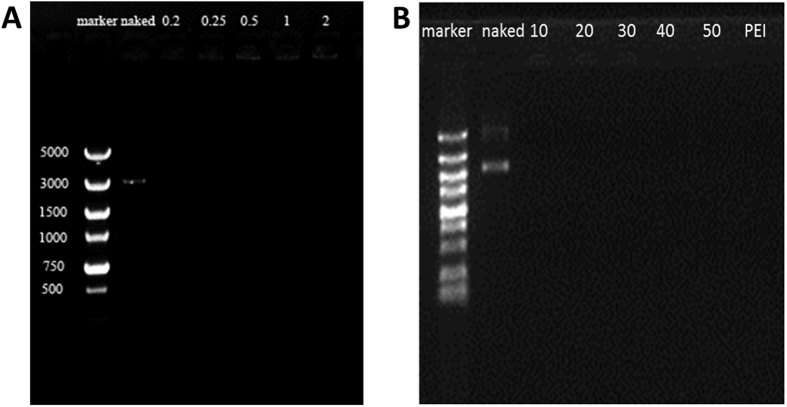
Agarose gel electrophoresis of polyplexes formed of PDAPEI and pDNA at various W/W ratios in the absence of (**A**) and presence (**B**) of 10% serum.

In gene delivery systems, condensing plasmid into nanoparticles is a prerequisite^26^ for protecting gene from degradation. The DNA binding capability of PDAPEI was measured by agarose gel electrophoresis. As shown in Fig. 3A, the migration of pDNA was completely blocked when PDAPEI/pDNA polyplexes were prepared at mass ratio of 0.2, which indicated that PDAPEI could wrap the plasmid DNA at this ratio. The agarose gel electrophoresis result proved that polycationic material PDAPEI can effectively condense plasmid DNA at a small ratio. Furthermore, capability of the self-assembled polymer in protecting the encapsulated plasmid against anionic polyelectrolytes and digesting enzymes was studied in the presence of 10% serum. Figure 3B showed that no released DNA was detected after 4 h incubation, illustrating the stability of polyplexes.

### Particle size, zeta potential and morphology measurements

**Figure 4:**
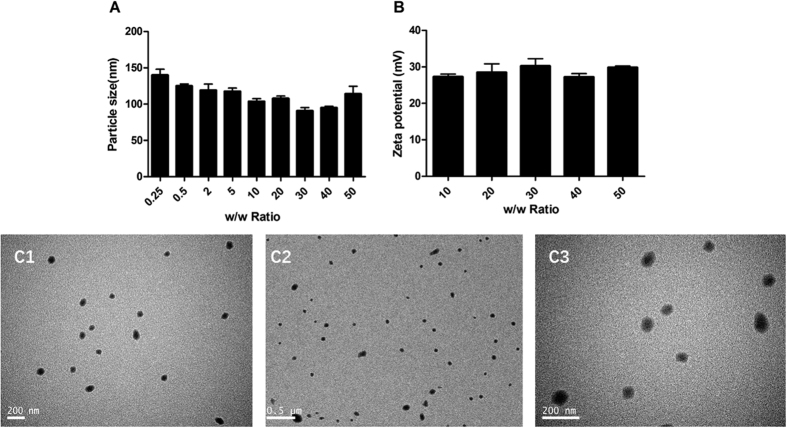
(**A**) Particle size and (**B**) zeta potential of PDAPEI/pDNA polyplexes of various w/w ratios at 37 °C. Data are shown as mean S.D. (n = 3). (**C**) TEM images of polyplexes formed of PDAPEI and pDNA at W/W ratio of 2.

Particle size and zeta potential of polyplexes not only reflect the DNA binding ability of polymer, but also affect the cell endocytosis and gene transfection efficiency^27^. In this case, particle size of PDAPEI/pDNA polyplexes was measured at different mass ratios ranging from 0.25 to 50. As shown in Fig. 4A, the average particle size of polyplexes gradually decreased from 140 nm to 90 nm with the increase of the polymer to DNA ratio ranging from 0.25 to 30. However, with the ratio increased from 30 to 50, the particle size did not decline as before but increased from 90 nm to 115 nm. The reason is discussed as follows: amino group of the polymer is required to be protonated firstly to generate positive charge, which can help condense DNA into nanoparticles. The capacity of condensing DNA depends on the surface electric charge density. At the same time, the polycationic material PDAPEI is pH-sensitive. Rising of pH is accompanied by the increase of mass ratios, which leads to the decrease of positive charge density. Within a certain range, with the rising of PDAPEI/pDNA ratio, the amino density as well as the capacity of condensing DNA increases. The increase of polymer concentration may raise the zeta potential, which accounts for the rising of particle size. All particle sizes were less than 150 nm, which was the maximum size for easy endocytosis^33^.

The zeta potential of polyplexes was shown in Fig. 4B. With the increase of w/w ratio ranging from 10 to 50, the zeta potential stabilized between 27 mV to 30 mV, which indicated that the system maintained in a stable state. Moreover, the positive charged particles can cross the cell membrane barrier because the cell membrane is negatively charged^33^,^34^.

The morphology of polyplexes was measured by transmission electron microscopy (JEOL JEM 2010 system). As shown in Fig. 4C, nanoparticle formed with PADPEI and pDNA had a spherical shape, uniform size and morphology. The particle size varied from 100 nm to 130 nm in average diameter, which was in correspondence with the result of particle size analysis.

### *In vitro* cell transfection

**Figure 5:**
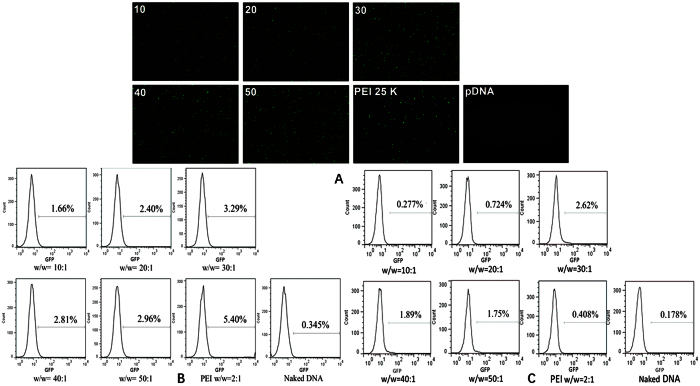
(**A**) *In vitro* GFP expression mediated by PDAPEI/pDNA polyplexes (the w/w ratio: 10,20,30,40,50, respectively). Naked DNA and the PEI 25 kDa/pDNA polyplexes (the w/w ratio: 2) were performed as controls. (**B**)Transfection efficiency in CT26 cells in the absence of serum defined as the percentage of GFP positive cells determined by flow cytometry. (**C**) Transfection efficiency in CT26 cells in presence of serum defined as the percentage of GFP positive cells determined by flow cytometry.

The ideal transport vector should possess high transfection efficiency and low cytotoxicity at the same time. In this study, we assessed the transfection efficiency of PDAPEI polymer using pDNA which encoded GFP reporter gene. Successfully transfected cells can express GFP reporter gene within 48 h. GFP-expressing cells were observed under fluorescence microscopy. As shown in Fig. 5A, in naked pDNA group almost no fluorescence was detected, which indicated that pDNA could hardly enter cells without being wrapped in polycationic materials. However, CT26 cells transfected by polyplexes formed with PDAPEI and pDNA at different ratios showed that the expression of GFP reporter gene was considerably high, especially at the ratios of 30, 40 and 50, which may be comparable to that of PEI 25 kDa/pDNA polyplexes.

In order to further determine the optimal ratio, we quantified the transfection efficiency with or without serum using flow cytometer. As shown in Fig. 5B, in the absence of serum, the transfection efficiency of PDAPEI/pDNA group (w/w = 30) was higher than the other four groups. Compared with PEI/pDNA group, the transfection efficiency of PDAPEI/pDNA group (w/w = 30) was almost the same, but significant improvement was achieved compared to pDNA group. However, in the presence of 10% serum (Fig. 5C), four groups (w/w = 20,30,40,50) exhibited higher transfection efficiency than PEI, especially for the mass ratio at 30. These results clearly indicated that PDAPEI had much higher serum tolerance than PEI 25 KDa. Also, higher polymer concentrations didn’t show better transfection results. It may be the reason that free PDAPEI has toxicity to CT26 cells and the toxicity may have consequences on the physiological function of cells and interfere the transfection of the polyplexes or the expression of GFP protein gene. Therefore, the cytotoxicity of polyplexes was tested below.

### *In vitro* cytotoxicity

**Figure 6:**
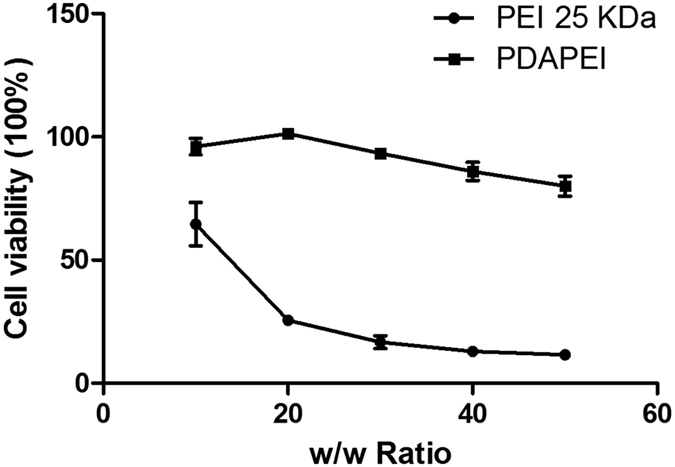
Cell viability (100%) of CT26 cells treated by PDAPEI/pDNA and PEI 25 kDa/pDNA polyplexes at different w/w ratios. Data are shown as mean ± S.D. (n = 6).

Cell Counting Kit (CCK-8) assay was used to measure the cytotoxicity of PDAPEI/pDNA polyplexes in CT26 cell line. As shown in Fig. 6, the cytotoxicity of PDAPEI/pDNA polyplexes was significantly lower than that of PEI/pDNA polyplexes. At different w/w ratios, PDAPEI/pDNA polyplexes remained low cytotoxicity with cell viability higher than 80%. However, the cell viability of those treated with PEI25 kDa/pDNA polyplexes dropped all the way from 60% to 20%. High molecular weight PEI contributes to high transfection, while also results in high cytotoxicity^22^. The low cytotoxicity of PDAPEI can be explained as follows: PDAPEI possesses small-molecule PEI as the basic unit and inserts 2,6-pyridinedicarboxaldehyde linker through forming imine linkage. PDAPEI can metabolize itself into low-molecular-weight PEI and 2,6-pyridinedicarboxaldehyde under acidic environment, which mainly leads to the decrease of cytotoxicity. Based on the above results, PDAPEI/pDNA at w/w ratio 30 was used as the optimal ratio in the following experiments.

### Intracellular uptake of PDAPEI/pDNA polyplexes

**Figure 7:**
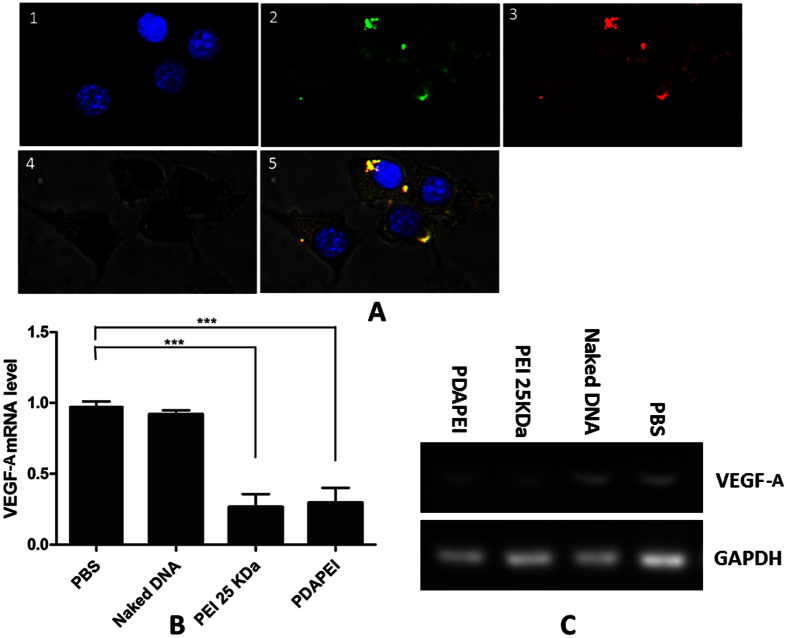
(**A**) Intracellular uptake of PDAPEI/pDNA polyplexes in CT-26 cells. For each panel, 1: nuclei stained by DAPI(blue); 2: endosomes stained by LysoTracker Green (green); 3: fluorophore Cy5 labeled PDAPEI/pDNA polyplexes (red); 4: bright field image; 5: overlay of 1, 2.3 and 4. (**B**) Relative levels of VEGF-A mRNA in CT-26 cells at 48 h after treatment were tested by qPCR. (**C**) Agarose gel electrophoresis of products of qPCR.

The intracellular transport of PDAPEI/pDNA polyplexes was determined using Confocal Laser Scanning Microscope (CLSM). As shown in Fig. 7A, the red fluorescence dots were induced from Cy5-labeled DNA and merged green fluorescence punctate, (The overlap image showed yellow fluorescence dots.), indicating that an endocytosis mechanism was anticipated in the intracellular uptake of PDAPEI/pDNA polyplexes. Meanwhile, a part of red dots was localized at the edge of the nucleus. These results proved that PDAPEI/pDNA polyplexes could be successfully delivered into cells, escaped from endosomes rapidly and readily transported into nucleus for effective gene expression.

### *In vitro* expression of VEGF-A

The expression level of VEGF-A mRNA in CT26 cells was determined by qRT-PCR. As shown in Fig. 7B, compared to no treatment group and naked DNA group, the expression level of VEGF-A mRNA in PDAPEI and PEI 25 K group was significantly lower. Naked DNA had a minor effect on down-regulation of VEGF-A level. However, PDAPEI/pDNA polyplexes performed an excellent gene silencing which could be equal to PEI 25 K group. Furthermore, the determination of qRT-PCR products using agarose gel electrophoresis obtained consistent results.

### *In vivo* anti-tumor studies

**Figure 8:**
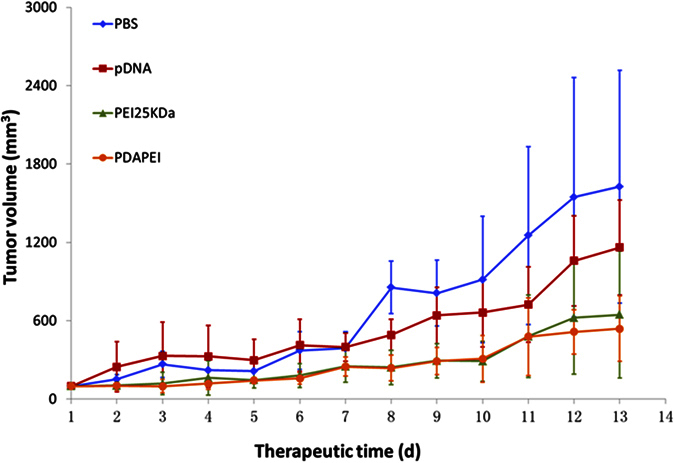
Tumor volume in CT26 subcutaneous tumor mice after intratumoral treatment with two vectors (PDAPEI and PEI 25 kDa)/DNA polyplexes. Results were represented as mean ± S.D. (n = 6). ***P < 0.001.

The angiogenesis suppression of PDAPEI/pDNA polyplexes was evaluated by monitoring tumor growth for 13 days in subcutaneous tumor models after intratumoral injection. As shown in Fig. 8, the tendency chart clearly showed tumor growth during the treatment period. The tumors in PEI group and PDAPEI group grew relatively slowly compared with saline group and pDNA group. However, from the seventh day on, the tumors in saline group and pDNA group experienced rapid tumor growth rate. On the contrary, the tumors in PEI group and PDAPEI group still kept a slow growth rate. The final average tumor volumes were as follows: 1627 mm^3^ in the saline group, 1160 mm^3^ in the pDNA group, 646 mm^3^ in the PEI group, and 539 mm^3^ in the PDAPEI group. The results showed that the tumor growth tendency in PDAPEI group was similar to that in PEI group or even lower.

**Figure 9:**
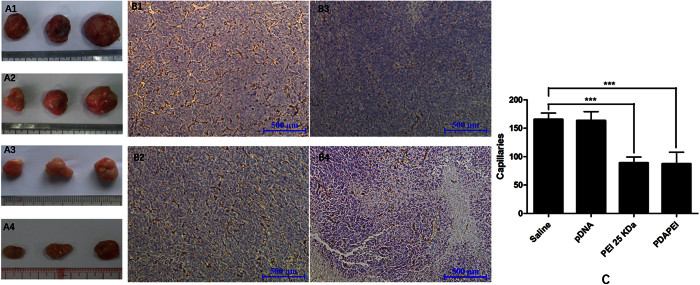
(**A**) Photograph of tumors in CT26 subcutaneous tumor mice after *in vivo* transfection mediated by PDAPEI and PEI 25 kDa vectors. (**B**) Representative images of vivo tumor sections assayed by immunohistology using CD31 antibody. (**C**) Quantification of CD31-positive microvessels in CT26 subcutaneous tumor mice treated with different formulations at the experimental end point (Day 13).

Subcutaneous tumors were peeled off and photographed after the mice were sacrificed. As shown in Fig. 9A, three representative tumors of each group were selected; the dark red capillaries on the surface of each tumor could be seen clearly. In saline group and pDNA group, the tumor volumes were significantly larger in size and the surfaces were relatively dark because of more capillaries when compared with PDAPEI group and PEI group. In immunohistochemistry analysis, CD31 staining was performed to display the existence of endothelial cells and to evaluate the angiogenesis in tumors, which could also illustrate the efficiency of anti-VEGF gene delivery by various formulations. As shown in Fig. 9B,C, the average capillary density of saline group, pDNA group, PEI group and PDAPEI group were: 165.6, 163.6, 89 and 87 respectively. Quantitatively, the capillary density of tumors in PDAPEI group was almost equal to that in PEI group but far lower than in other two groups. Although in *in vitro* examination, PDAPEI had higher serum tolerance than PEI, the cytotoxicity of PEI might contribute to the suppression of tumor growth. Collectively, these results persuasively implied that the anti-VEGF gene was successfully transported into cells by polycationic material delivery systems.

**Figure 10:**
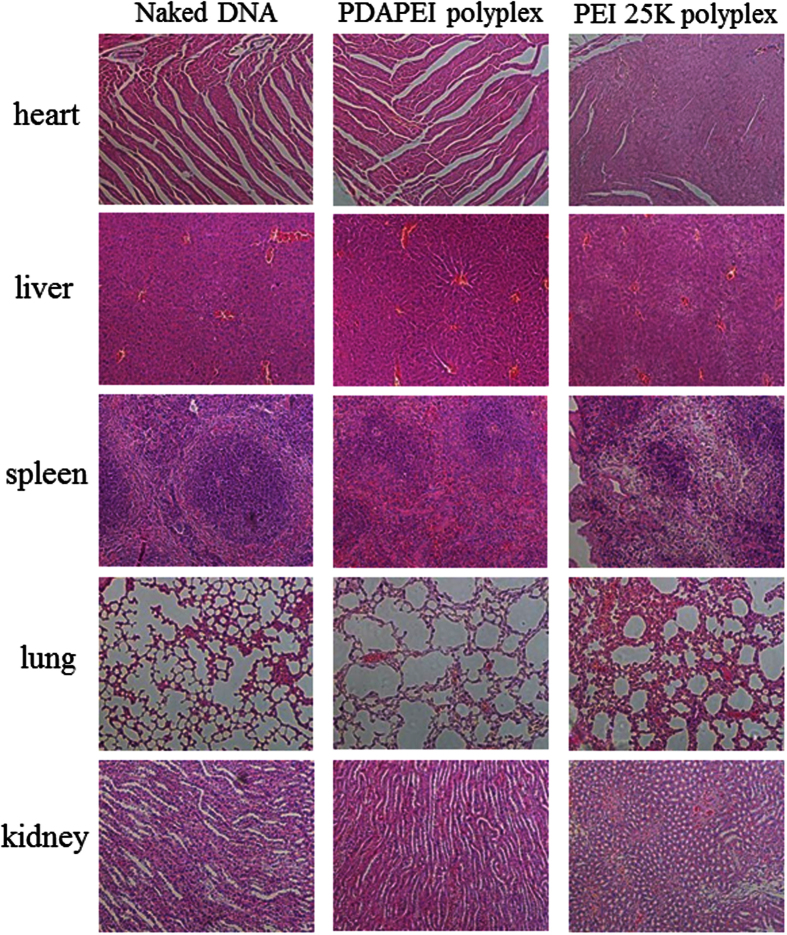
Histological sections of organs stained with H&E in naked DNA, PDAPEI and PEI 25 K treated mice after 13 days. Data are representative of 6 mice.

The *in vivo* cytotoxicity of PDAPEI was shown in Fig. 10. Compared with naked DNA group, the PDAPEI polyplexes showed a negligible toxicity after intra-tumoral injection. However, positive control PEI 25 K caused inflammation and apoptosis in almost five organs. These results suggested that compared to PEI 25 K PDAPEI had relatively good tissue compatibility against main organs after intra-tumoral injection.

### Innate immune response

**Figure 11:**
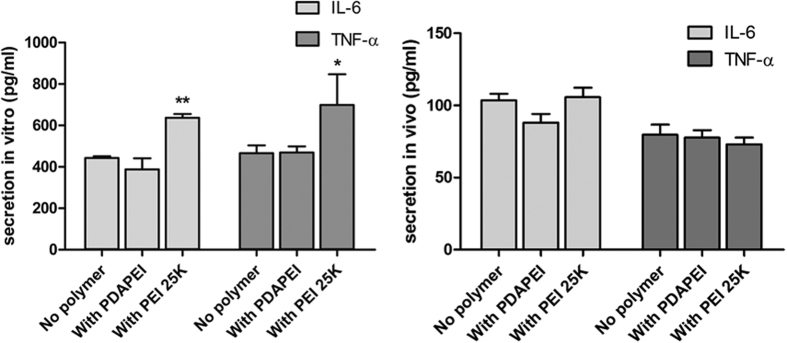
The secretion levels of IL-6 and TNF-α treated with naked DNA, PDAPEI polyplexes and PEI polyplexes from murine RAW264.7 macrophage cells and Balb/c mice.

In order to measure the secretion of IL-6 and TNF-α which generally represent the induction of the innate immune response, murine RAW264.7 macrophage cells and Balb/c mice were treated with naked DNA, PDAPEI polyplexes and PEI polyplexes. As shown in Fig. 11, while the levels of secreted IL-6 and TNF-α from cells treated with PEI polyplexes were increased 1.5-fold when compared to that with naked DNA, there was no significant induction of IL-6 and TNF-α from cells treated with PDAPEI polyplexes. Moreover, in *in vivo* experiments, no significant difference was detected in IL-6 and TNF-α level of mice treated with or without polymers. These results indicate that the designed polymer PDAPEI does not stimulate the innate immune system with the tested dosage.

## Conclusion

In summary, the PDAPEI/pDNA polyplexes showed an equivalent effect of inhibition of tumor angiogenesis and lower cytotoxicity compared to PEI 25 KDa/pDNA polyplexes. This improvement may be due to several reasons: (1) This polycationic material PDAPEI can wrap and condense DNA into nanoparticles to protect it from degradation. (2) PDAPEI can help endosomal escape of plasmid DNA by proton sponge effect and then release DNA in cytoplasm. (3) The anti-VEGF gene we have constructed can silence target gene effectively. (4) PDAPEI can metabolize itself into small-molecular PEI and 2,6-pyridinedicarboxaldehyde under acidic environment, which have negligible toxicity.

## Additional Information

**How to cite this article**: Che, J. *et al*. Biologically responsive carrier-mediated anti-angiogenesis shRNA delivery for tumor treatment. *Sci. Rep.* **6**, 35661; doi: 10.1038/srep35661 (2016).

## Figures and Tables

**Figure 1:**
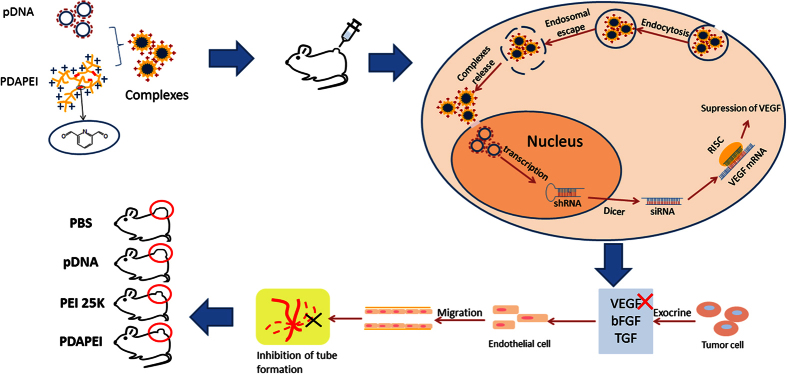
Schematic illustration shows the role of PDAPEI in tumor angiogenesis. The PDAPEI can condense DNA into nanoparticles, then, efficiently deliver DNA into the nucleus and the produced RISC can directly target the VEGF mRNA. Reducing the amount of VEGF secreted by tumor cells inhibits the tube formation, which contributes to the suppression of tumor growth.
